# Computer Simulations Imply Forelimb-Dominated Underwater Flight in Plesiosaurs

**DOI:** 10.1371/journal.pcbi.1004605

**Published:** 2015-12-18

**Authors:** Shiqiu Liu, Adam S. Smith, Yuting Gu, Jie Tan, C. Karen Liu, Greg Turk

**Affiliations:** 1 School of Interactive Computing, Georgia Institute of Technology, Atlanta, Georgia, United States of America; 2 Nottingham Natural History Museum, Wollaton Hall, Nottingham, United Kingdom; Indiana University, UNITED STATES

## Abstract

Plesiosaurians are an extinct group of highly derived Mesozoic marine reptiles with a global distribution that spans 135 million years from the Early Jurassic to the Late Cretaceous. During their long evolutionary history they maintained a unique body plan with two pairs of large wing-like flippers, but their locomotion has been a topic of debate for almost 200 years. Key areas of controversy have concerned the most efficient biologically possible limb stroke, e.g. whether it consisted of rowing, underwater flight, or modified underwater flight, and how the four limbs moved in relation to each other: did they move in or out of phase? Previous studies have investigated plesiosaur swimming using a variety of methods, including skeletal analysis, human swimmers, and robotics. We adopt a novel approach using a digital, three-dimensional, articulated, free-swimming plesiosaur in a simulated fluid. We generated a large number of simulations under various joint degrees of freedom to investigate how the locomotory repertoire changes under different parameters. Within the biologically possible range of limb motion, the simulated plesiosaur swims primarily with its forelimbs using an unmodified underwater flight stroke, essentially the same as turtles and penguins. In contrast, the hindlimbs provide relatively weak thrust in all simulations. We conclude that plesiosaurs were forelimb-dominated swimmers that used their hind limbs mainly for maneuverability and stability.

## Author Summary

Plesiosaurs are an extinct group of Mesozoic marine reptiles with a global distribution that spans 135 million years. They maintained a unique body plan with two pairs of large wing-like flippers throughout their long evolutionary history, but how plesiosaurs swam has remained a topic of debate for almost 200 years. We address the question of how plesiosaurs swam using a digital, three-dimensional, free-swimming model of a plesiosaur in a simulated fluid. We performed thousands of simulations under different parameters to investigate possible plesiosaur swimming patterns. Our simulations show that the forelimbs provide the majority of thrust, and that the thrust from the hindlimbs is weak. The plesiosaur swims primarily with its forelimbs using an underwater flight stroke, essentially the same as turtles and penguins.

## Introduction

Plesiosaurians (= plesiosaurs) are an extinct group of highly derived predatory marine reptiles with a global distribution that spans 135 million years from the Early Jurassic to the Late Cretaceous. During their long evolutionary history **[** 1], plesiosaurs maintained a unique body plan with two pairs of large wing-like flippers—a unique adaptation in the animal Kingdom [2, 3, 4, 5, 6, 7, 8, 9]. Although plesiosaurs were a key component of Mesozoic marine ecosystems, there are no extant ‘four-winged’ analogues to provide insights into their behavior or ecology, and their locomotion has remained a topic of debate since the first complete plesiosaur skeleton was described in 1824 [10]. Key areas of controversy have concerned the most efficient biologically possible limb stroke, and how the four limbs moved in relation to each other. Previous studies of plesiosaur locomotion have endorsed a variety of strokes and gaits. Stroke hypotheses include a rowing stroke akin to the oar of a boat [4], a flight stroke [5, 3] akin to penguins and turtles [11, 12], and a modified flight stroke [6] akin to sea lions [13]. Study of plesiosaurian musculature does not rule out either rowing or flight strokes [14]. Gait hypotheses include synchronous motion with all four limbs moving in phase [15], asynchronous motion with the forelimbs and hindlimbs out of phase [12, 7], and semi-synchronous motion [3]. Some authors have proposed that the hindlimbs provided most of the propulsion [16], whereas others suggest that the forelimbs provided the majority of thrust [8]. The question has been approached experimentally using robotics [17] and human swimmers with fabricated paddles [3]. These studies, although informative, are limited because they do not deal with accurate representations of the plesiosaur form. There is therefore still no consensus on how plesiosaurs swam, especially how they moved all four limbs relative to each other.

Our approach uses computer simulation to address the question of how plesiosaurs swam using a three-dimensional plesiosaur model in a simulated fluid. The computational model explores a given range of joint motion to discover the swimming stroke and gait that propels the creature forward the greatest distance. There are two main advantages to using computer simulation to investigate swimming motions of plesiosaurs. First, we can construct a digital representation of the body that accurately matches the known body and limb shapes of a particular species. Second, we can run thousands of trials with different strokes and gaits to explore the space of possible swimming motions. We use an optimization method to search for the highest quality motion for a given range of joint angles. Computer simulation has been used to investigate the motions of several types of modern-day swimmers, including fish [18–21], tadpoles [22], and copepods [23]. To our knowledge, our work is the first use of computer simulation to study the swimming of plesiosaurs.

## Results/Discussion

### The plesiosaur model

**Fig 1:**
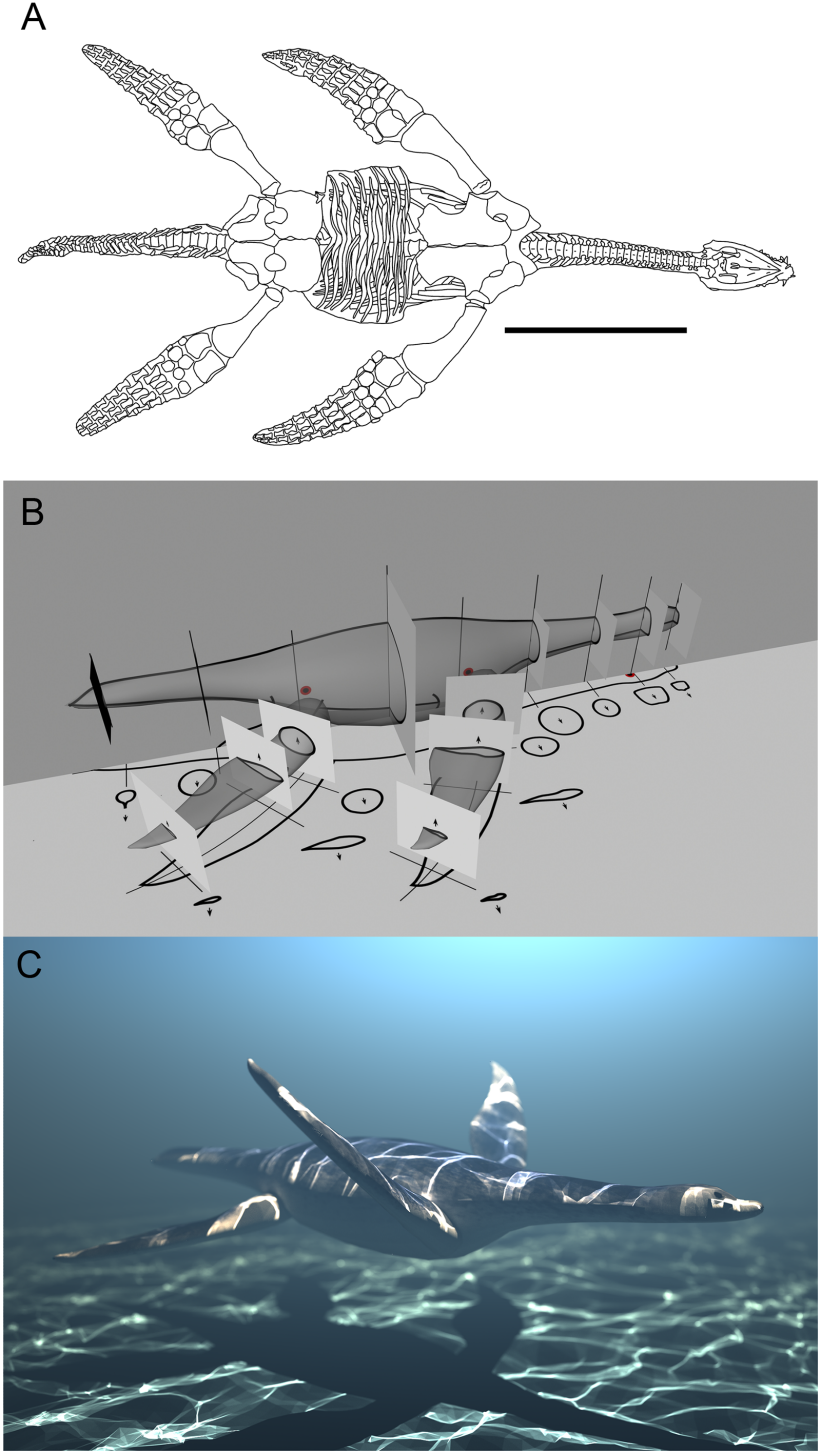
3D model construction. (A) Illustration of the holotype of *Meyerasaurus victor* (SMNS 12478) (Scale bar = 1m). (B) The three-dimensional model was based on a series of two-dimensional cross sections. (C) Static computer rendering of the final three-dimensional model of *Meyerasaurus victor* used in our study.

We constructed a life-sized plesiosaur model based on *Meyerasaurus victor*, a small (3.35 meters long) taxon from the Lower Jurassic of Germany, because it is known from an almost complete articulated skeleton (SMNS 12478) with all four limbs preserved in their entirety [24] (Fig 1A). In addition, *Meyerasaurus* possesses a generalized morphotype among plesiosaurs, with a moderately long neck, so it can be considered representative of the clade Plesiosauria as a whole, which contains long- and short-necked morphotypes [2]. The shape and proportions of the model were based directly on the skeleton, with three-dimensional data and soft tissues (e.g. muscles, cartilage, integument) reconstructed based on evidence from other taxa.

Two dimensional cross-sectional data for the body was estimated by tracing around the skeleton in the horizontal plane. Since the holotype of *Meyerasaurus* is dorsoventrally compressed, information from other plesiosaur specimens was used to estimate cross sections in the vertical plane [25] (Fig 1B). Transverse cross-sections of the limbs were derived from the three-dimensionally preserved propodials of the closely related *Rhomaleosaurus thorntoni* [26]. The restored limbs are cambered hydrofoils in section with a narrower postaxial trailing edge. The postaxial edge of the limb was extended beyond the osteological anatomy to reflect fossil evidence for a soft tissue trailing edge in this region in *Seeleysaurus guilelmiimperatoris* [27] and *Hydrorion brachypterygius* [28]. The tail of the model was reconstructed with a short dorsally expanded mediolaterally compressed fin based on evidence from several plesiosaurian taxa, including the sister taxon to *Meyerasaurus*: *Rhomaleosaurus* [29]. The virtual model was constructed in Maya, a widely used CAD tool. First, the two-dimensional cross sections were aligned in the horizontal and vertical planes (Fig 1B). Second, the model was constructed using these sections as a reference. Since our grid-based fluid simulator cannot detect features under 67 mm thick, the thinnest parts of the anatomy, such as the trailing edge of the limbs, were artificially dorsoventrally thickened. For simplicity, the density of the plesiosaur model is identical to that of the fluid (i.e. it is neutrally buoyant), and therefore our simulations do not take into account possible variation in buoyancy along the body of the animal due to air-filled lungs, or gastroliths [30]. The life size final constructed plesiosaur model is 3.35 meters long from head to tail (Fig 1C). An alternative bulkier *Meyerasaurus* model with 50% greater soft-tissue mass around the base of the limbs was also created to test the effect of a bulkier body outline.

**Fig 2:**
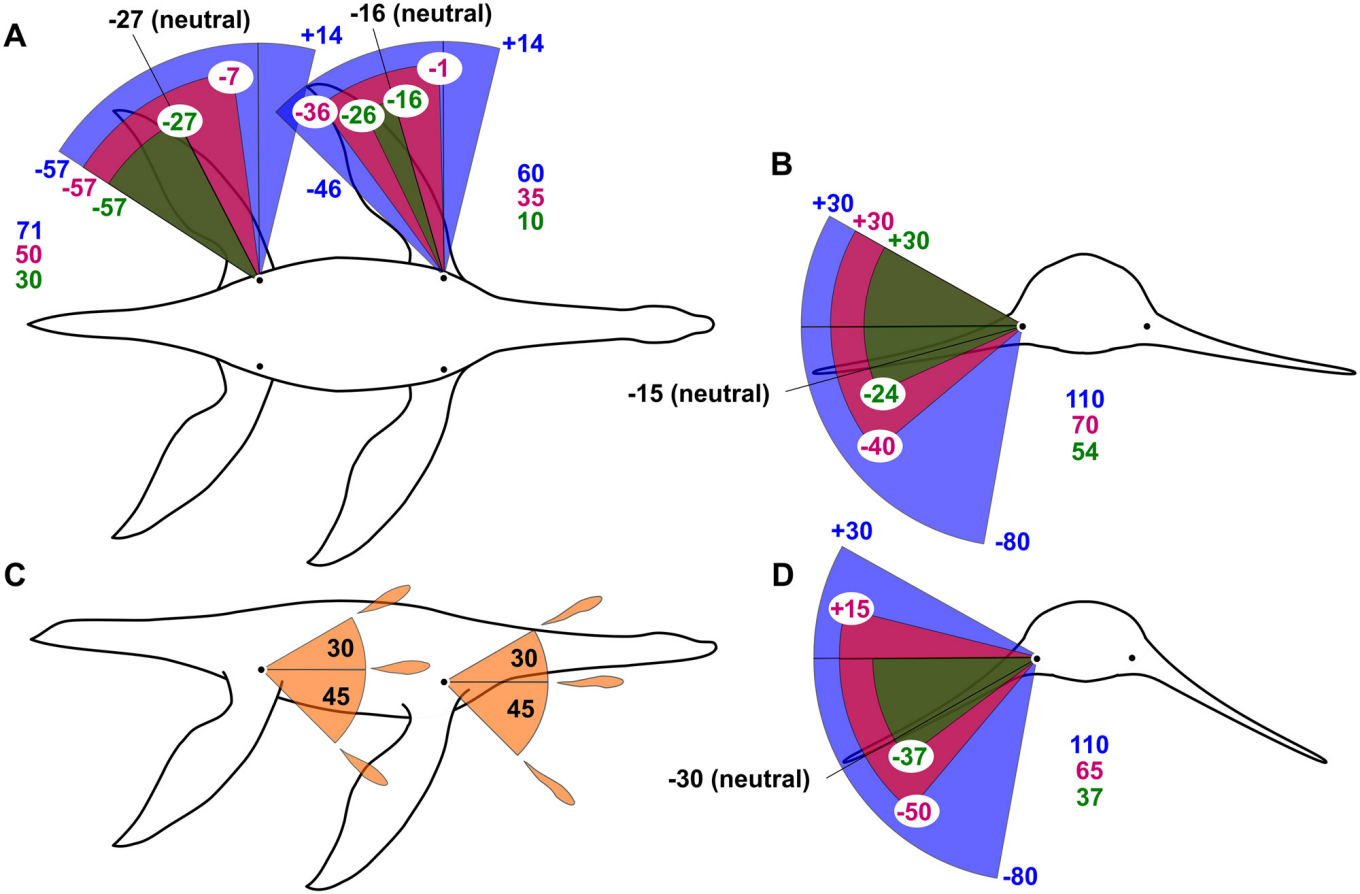
Outlines of the *Meyerasaurus* model showing the available ranges of motion in each simulation. (A) dorsal view showing the anteroposterior ranges of motion, (B) transverse section through the pectoral region showing the dorsoventral ranges of the forelimbs, (C) lateral view showing the degree of rotation available in all simulations (identical in all simulations), (D) transverse section through the pelvic region showing the dorsoventral ranges of the hindlimb. There are three contrasting ranges of motion: ‘narrow’ (green), ‘medium’ (pink), and ‘wide’ (blue). Note that the angles in (A) are measured from a line drawn perpendicular to the long axis of the body relative to the long axis of the propodials (not the limb as a whole, which curves posteriorly), and the angles in parts (B), (C) and (D) are measured from the horizontal relative to the long axis of the propodials.

Our physics simulator (based on previous methods [31]) represents the plesiosaur as a collection of rigid body parts that meet at points of articulation. Specifically, we model the body (torso, neck, and tail) as one rigid component and the four limbs as additional rigid parts. In life, the plesiosaur torso was a rigid structure, since a sturdy trunk is a prerequisite for purely paraxial underwater locomotion. The neck and tail in plesiosaurs were flexible in life to variable degrees [32], so they may have had a relatively minor role in propulsive locomotion. However, since our focus is on the question of limb-based propulsion in a four-winged paraxial swimmer, we kept the neck and tail immobile in the simulation to allow us to focus solely on the movement of the limbs. Each limb is joined to the body by a three-degree-of-freedom joint (Fig 2). These four joints can be actuated internally to generate motion. Each swimming motion is represented as a sinusoidal function at each joint degree of freedom, and the actuators track these desired motions by applying torques at the joints. In turn, the motion of the body and limbs affects the simulated fluid that surrounds the animal. Our simulator resolves the motion of the animal body and the fluid simultaneously, so that the final motion is due to the interaction between the body and the fluid. This is in contrast to studio-created computer animation, where the motion of the animal through the fluid is scripted by an artist, and may not obey the governing laws of physics.

### Stroke modeling and ranges of joint motion

To study the forward swimming motion across a wide range of periodic swimming strokes of the limbs, we require a motion representation that is expressive but that is also biologically plausible. We decouple the degrees of freedom at a joint into a dorsal/ventral component, an anterior/posterior component, and a pronate/supinate component (the rotational angle of the limb). We specify the limb motions of the plesiosaur by describing sinusoidal patterns for each of the three degrees of freedom at a given joint. The limb motion for a given component is specified by three values: the minimum and maximum value of the sinusoid, and the phase of the sinusoid. We use the same frequency (0.5 Hz) for all of the sinusoids across all of the limbs. Since the motion for each degree of freedom is given by three values (maximum and minimum range, and phase), nine numbers fully describe the motion of a single limb. We assume that the left and right limbs move in synchrony while the animal is swimming straight, as is the case for penguins, sea lions, marine turtles, and nothosaurs [33]. However, we specifically allow the front and back limbs to follow different patterns of motion: the minimum and maximum angles, and the phase of the sinusoid for the front and back limbs can be set differently. This allows us to test, for instance, the possibility that the front and back limbs move together or with offset phases. To specify both front and back limb sinusoidal motion, we require a total of 18 parameter values. Table 1 shows the optimized minimum/maximum ranges for all limbs, as well as the average travelling velocity and distance traveled for all of our experiments.

**Table 1:** Joint ranges. Table showing the joint ranges used in each optimization, and the speed and distance travelled by the plesiosaur. The percentages given in brackets indicate the proportion of the available range used in each optimization.

Joint Range	Forelimb Dorsoventral (degrees)	Forelimb Anteroposterior (degrees)	Forelimb Rotation (degrees)	Hindlimb Dorsoventral (degrees)	Hindlimb Anteroposterior (degrees)	Hindlimb Rotation (degrees)	Distance (m)	Speed (m/s)
All Limbs—Narrow	29/-23 (96%)	-16/-21 (50%)	42/-28 (93%)	-2/-27 (68%)	-34/-53 (63%)	37/-5 (56%)	0.63	0.32
All Limbs—Medium	29/-39 (97%)	-4/-20 (45%)	41/-26 (89%)	10/-48 (89%)	-10/-52 (84%)	42/-12 (72%)	0.92	0.46
All Limbs—Wide	27/-80 (97%)	13/-43 (93%)	45/-20 (87%)	9/-71 (72%)	-18/-47 (41%)	16/-16 (42%)	1.57	0.79
Forelimbs—Narrow	29/-23 (96%)	-16/-21 (50%)	44/-28 (96%)				0.59	0.3
Forelimbs—Medium	29/-39 (97%)	-1.5/-20 (53%)	44/-26 (93%)				0.83	0.42
Forelimbs—Wide	-30/-79 (99%)	13/-45 (97%)	43/-21 (85%)				1.68	0.84
Hindlimbs—Narrow				-16/-22 (16%)	-27/-56 (97%)	44/-2 (61%)	0.14	0.07
Hindlimbs—Medium				13/-49 (95%)	-7/-45 (76%)	44/-14 (77%)	0.26	0.13
Hindlimbs—Wide				29/-79 (98%)	-6/-29 (32%)	39/-29 (77%)	0.56	0.28

**Fig 3:**
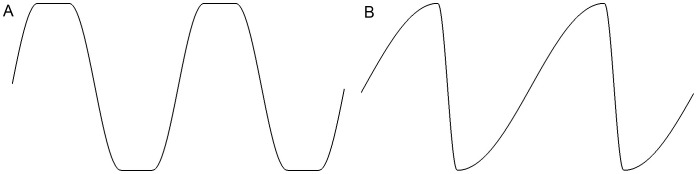
Modified sinusoids. (A) A modified sinusoid that is flat for a given duration, to allow the limb rotation to remain fixed during part of a stroke. (B) Another modified sinusoid that allows for time asymmetry between the downstroke and the upstroke of a limb.

Although using sinusoidal motions of various angle ranges and phases gives a wide range of possible swimming strokes, the motions of modern-day swimming animals depart from pure sinusoidal motion in at least two ways. Animals such as penguins that use an underwater flight stroke [12] hold the angle of rotation steady during the downstroke, quickly rotate the limb at the bottom of the stroke, and then hold the angle of rotation steady again during the upstroke. We allow for this possibility by using one additional degree of freedom for the rotation of a limb that specifies the duration of a motionless interval during which the limb maintains a zero rotational velocity. This interval can be set to zero, which indicates pure sinusoidal motion, or it can be non-zero to hold the angle of rotation steady through a portion of the stroke (Fig 3A). This gives us two additional motion parameters, one for the forelimbs and one for the hindlimbs. We also allow for the possibility that the animal’s downstroke and upstroke take different amounts of time (Fig 3B). This is in recognition of the observation that plesiosaurs may have had stronger musculature governing the downstroke of their limbs [7, 9, 14]. We add one more degree of freedom for each sinusoid to specify its degree of time asymmetry. This gives us six additional motion degrees of freedom, bringing the total number of parameters that describe a periodic swimming motion to 26. Table 2 shows the time asymmetry as well as motionless portion in pronate/supinate direction optimized in all of our experiments.

**Table 2:** Parameters for modified sinusoids. The downstroke/upstroke time asymmetry allows the downstrokes and upstrokes to take different amount of time. A value larger than 0.5 means faster ventral, pronate and posterior stroke, while values less than 0.5 means faster dorsal, supinate and anterior stroke. The rotation motionless interval allows the limb to hold the angle of rotation fixed during the stroke and rotate quickly at the top or bottom. The larger the interval, the longer the fixed interval and the quicker the rotation.

	Downstroke/Upstroke Time Asymmetry						Rotation Motionless Interval	
	Forelimb Dorsoventral	Forelimb Rotation	Forelimb Anteroposterior	Hindlimb Dorsoventral	Hindlimb Rotation	Hindlimb Anteroposterior	Front Limb Motionless Interval (seconds)	Hind Limb Motionless Interval (seconds)
All Limbs—Narrow	0.65	0.65	0.56	0.61	0.62	0.54	1.39	1.16
All Limbs—Medium	0.64	0.60	0.52	0.64	0.61	0.52	1.39	1.02
All Limbs—Wide	0.65	0.62	0.58	0.56	0.59	0.59	1.2	0.82
Forelimbs—Narrow	0.65	0.56	0.56				1.40	
Forelimbs—Medium	0.64	0.59	0.58				0.56	
Forelimbs—Wide	0.65	0.64	0.53				1.07	
Hindlimbs—Narrow				0.50	0.65	0.50		1.05
Hindlimbs—Medium				0.50	0.57	0.50		0.94
Hindlimbs—Wide				0.52	0.51	0.50		1.12

Plesiosaur limbs contain a single mobile joint located between the propodial and the girdle: the glenohumeral joint in the forelimb and the acetabulum-femoral joint in the hindlimb. The articulation points for these joints in the model are located in the anatomically-correct positions (Fig 2). Neutral limb positions were derived from existing estimates for *Plesiosaurus* sp. [3]. In the forelimb the neutral position is -15 degrees from the horizontal and -16 degrees from a line drawn perpendicular to the long axis of the body. In the hindlimb the neutral position is -30 degrees from horizontal and -27 degrees from a line drawn perpendicular to the long axis of the body (Fig 2). In forelimb only and hindlimb only optimizations, the static limbs are locked into these neutral positions and the active limbs are initiated in the fully abducted positions. While the available range of rotation along the long axis of the limb was identical in all limbs and optimizations: up to 30 degrees supination and 45 degrees pronation (Fig 2C), we tested three different ranges of motions in the dorsal/ventral and anterior/posterior directions (Fig 2A–B,D). The degree of freedom in the joints of living plesiosaurs was dependent on the extent and thickness of the cartilage that covered the head of the propodials and lined the glenoid and acetabulum. To account for possible differences in cartilage thickness and to investigate stroke efficiency and gait under different specified parameters, we performed optimizations under three different ranges of joint freedom: ‘narrow’, ‘medium’, and ‘wide’ (Fig 2). The narrow range was taken directly from conservative estimates of degrees of freedom in *Plesiosaurus* sp. [3]. The wide range provides an expanded degree of freedom that possibly exceeds the biologically possible range in the living animal, and the medium range represents a realistic compromise between the conservative narrow range and generous wide range.

In life, rotation of these joints was complicated, but for simplicity, they pivot around a single point in the model. Although there are no additional mobile joints in plesiosaur limbs, cartilage and tendons would have allowed dorsoventral flexibility and twisting along the long axis of the limb [5]. This could have resulted in an increased range of motion at the tip of the limb compared to the range of motion at the joint, and may have affected water flow and minimized drag. One limitation of our method is that it does not currently replicate flexibility of this kind—the limbs are rigid elements in our simulations. We accounted for this, in part, by providing simulations with wider ranges. To fully address limb flexibility would require an entirely different simulator that uses the finite element method to allow limb deformations. This would also require a different approach to solid/fluid coupling in the simulator.

### Optimization for different joint ranges

Since a single swimming motion requires the specification of many different sinusoidal parameters (26, as described above), we use numerical optimization to explore the range of possible plesiosaur swimming motions. Specifically, we use the sample-based method called Covariance Matrix Adaptation [34] which has been used to investigate eel swimming [19] and animal walking gaits [35]. Our optimization process runs several thousand different simulations with different joint motions, narrowing in on the set of motions that produces the fastest swimming motion. Note that for such a large parameter space, CMA is not guaranteed to find the global optimum. However, we observed only small variations in the final results of different CMA runs with the same parameter settings.

**Fig 4:**
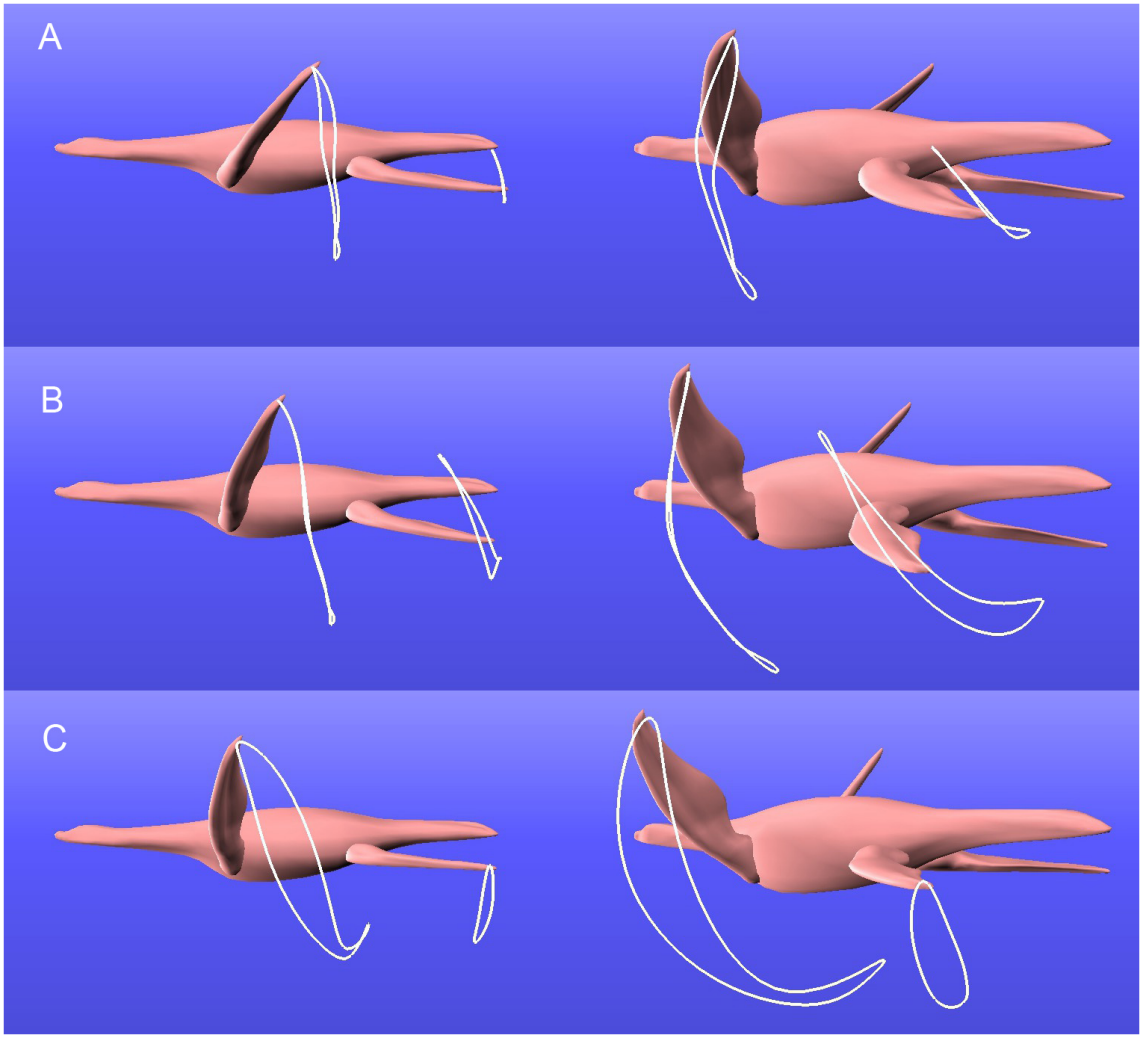
Tip traces of the most efficient swimming strokes for each of the three ranges. (A) narrow, (B) medium, and (C) wide. The best forelimb stroke for the narrow and medium ranges is underwater flight, whereas the best stroke for the wide range is modified flight.

Fig 4 shows the resulting optimal swimming motions for each of the three joint ranges, where both the front and back limbs move in a manner that best propels the plesiosaur forward. The white paths in the figure show the distal tip traces. (See the two accompanying S1 and S2 Videos for the detailed motions.) The best strokes for the forelimbs in both the narrow and medium range is an underwater flight motion, in which the limbs move primarily in the dorsoventral direction, and only rotate at the top and bottom of the stroke (Fig 4A and 4B). This pure flight stroke has been suggested as the most likely swimming stroke for plesiosaurs based on several anatomical lines of evidence [5]. In contrast, our optimization determined that the best forelimb stroke for the large range of joint motion is a modified U-shaped flying stroke (Fig 4C). This is similar to a flight stroke, but with more posterior motion during the power stroke and with a partially feathered recovery, and has also been proposed for plesiosaurs [6, 15, 8]. Note, however, that our optimizations suggest that this modified flight stroke is only plausible under the most liberal of joint range assumptions, and such a wide range of motion is considered biologically impossible [3]. None of our optimizations produced a substantial rowing stroke, as had been suggested by early researchers [4].

S1 Video shows the highest quality swimming motions from each of our optimization runs. To produce each of these video segments, we re-computed the simulation that corresponded to the highest quality motion sample that was found during the given optimization run. There are nine motion clips in this video, corresponding to the wide, medium and narrow ranges of limb joints, with motion from both pairs of limbs, just the forelimbs, and just the hindlimbs. As in the optimization runs, the model plesiosaur is initially at rest and then begins to move. During the first stroke, the model plesiosaur sometimes turns upwards, but then moves straight during subsequent strokes. For this reason we do not include the motion of the first stroke in our quality assessment (described in detail later).

The simulations used to make these videos not only provide the motion of the plesiosaur model, but also give us the velocity field for the simulated fluid at each simulation time step. This allows us to show not only the plesiosaur motion, but also the accompanying motion of the water. In each video clip, we show the motion of the fluid using particle traces (particle trajectories over the last few time steps) from randomly positioned particles in the virtual fluid. These massless particles are passively advected through the fluid. The particle traces are drawn only at locations with large vorticity (greater than 1.5 s^-1^) to concentrate them at regions of interest.

S2 Video shows the plesiosaur limb stroke motions from these same nine simulations, and provide traces of the tips of each moving limb. These motion clips allow a clearer picture of the limb motions relative to the body. In these video clips, the camera moves together with the body so that the body appears to be stationary. Two views are provided for each motion: a lateral view and a posterolateral view. For clarity, particle traces have been omitted in these video clips.

**Fig 5:**
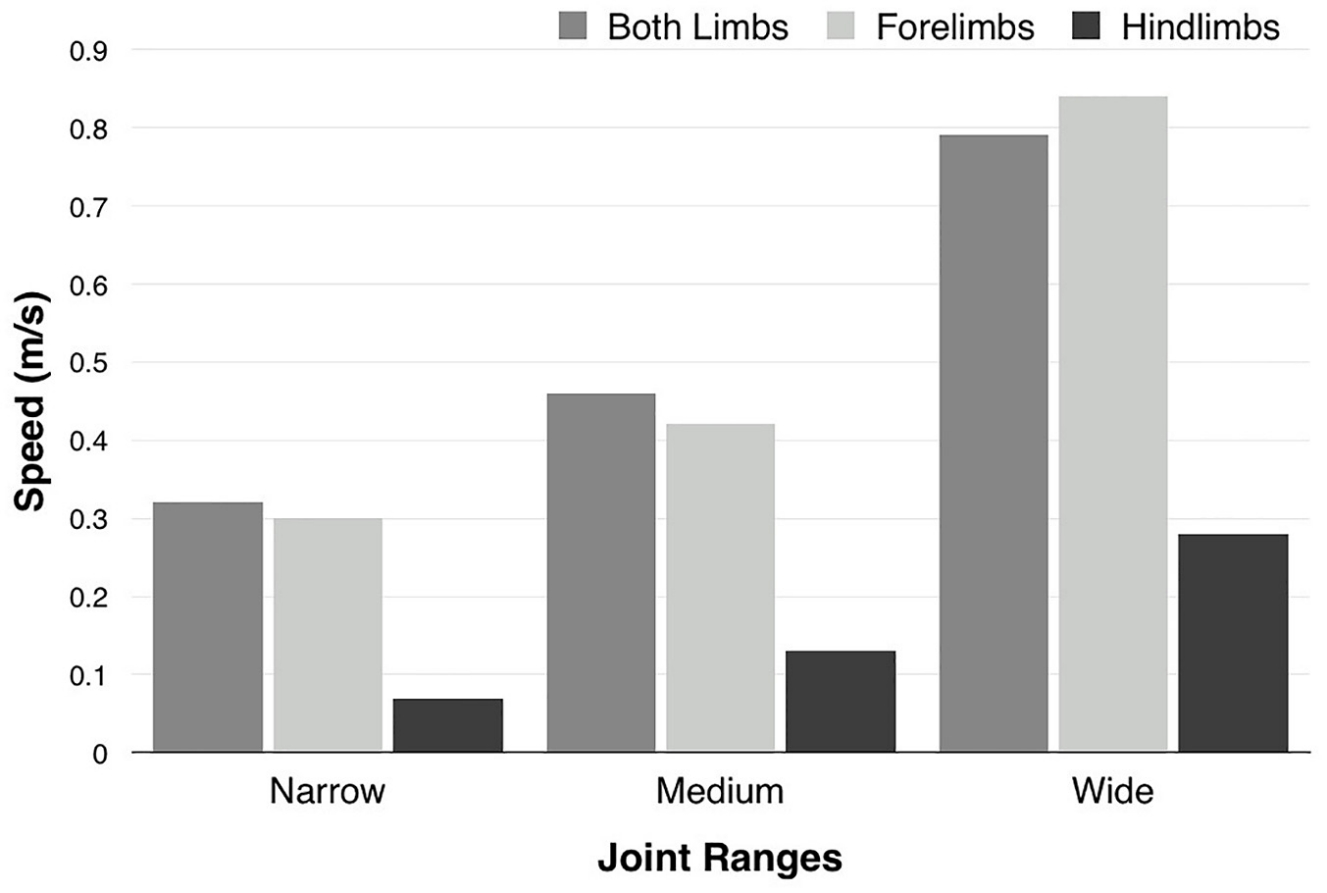
Swimming speeds. The speed of the best motions that were found by the optimization for each of the joint ranges (narrow, medium, wide). Note that the speed in forelimb-only optimizations is similar to the equivalent all-limbs optimizations, while hindlimb-only optimizations are substantially slower.

Fig 5 shows the best swimming speeds for each joint range. These swimming speeds are similar to prior estimates of an optimum swimming speed of 0.48 m/s for *Meyerasaurus* [36]. Because all of the strokes have a two second period, it is to be expected that the larger ranges of motion result in a faster speed, so no conclusions should be drawn from the relative speeds between the three different ranges of joint motion.

### Locomotory gait

There are several potential relative motions between the forelimbs and hindlimbs during swimming. One proposal is that asynchronous motion is the most likely way to produce continuous forward motion [9]. Synchronous (and semi-synchronous) motion has been deemed more likely by other researchers [15, 8, 3]. Our optimizations provided no clear answer to this question, which is significant in itself. In the narrow range the limbs move asynchronously, in the medium range they move semi-synchronously, and in the wide range they move synchronously.

In order to deduce the separate contributions of the forelimbs and hindlimbs to propulsion we performed optimizations with forelimb-only motion and hindlimb-only motion. The results were strikingly consistent across all three joint ranges. The forelimb-only strokes from optimization were roughly as fast as the best gaits resulting from optimizations using all four limbs (Fig 5). In contrast, the hindlimb-only strokes from optimization provide a much slower forward motion, even for the widest joint range. This inability of the hindlimbs to generate thrust explains the lack of consistency in the relative motions of the forelimbs and hindlimbs in our optimization results. It does not matter whether the plesiosaur moves its hindlimbs in or out of phase with the forelimbs, since neither strategy will contribute substantially to forward motion. Our optimization results imply that plesiosaurs were forelimb-dominated swimmers, and that the hindlimbs contributed little to their forward motion. This is consistent with trace-fossil evidence for forelimb-dominated locomotion in nothosaurs [33]. It also corroborates other studies that concluded that the hindlimbs were used primarily for steering and stabilization during swimming [8], and that two-flippered gaits serve well for low-cost cruising [17]. The plesiosaur hindlimbs, despite their wing-like shape and large size, played a diverse role in locomotion, but a relatively minor role in propulsion. They supplemented the forelimbs, which were the primary propulsive organs, by enhancing maneuverability and stability, possibly in conjunction with the tail [29].

**Fig 6:**
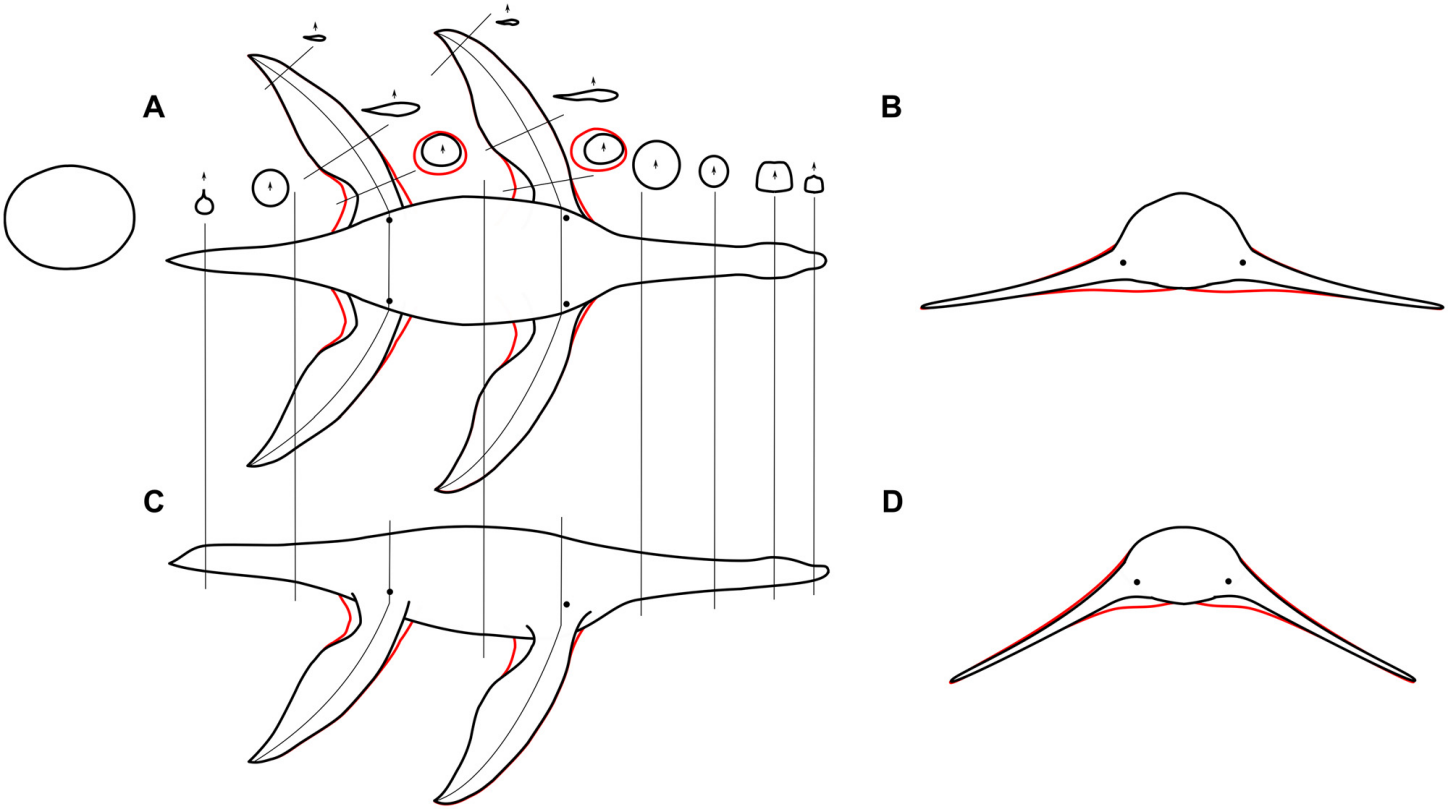
Two body models. To test the effect of muscle bulk at the base of the limbs, we built two different body models. The slim model is shown in black, and the modifications to increase the muscle bulk are indicated in red. (A) dorsal view with transverse sections through the neck, body, tail, and flippers, (B) transverse section through the pectoral region, (C) lateral view, (D) transverse section through the pelvic region.

To investigate the effect of small body changes to swimming speed, we constructed an alternative version of the *Meyerasaurus* model with 50% greater ‘muscle mass’ around the bases of the limbs. Fig 6 shows the original and modified body shapes. We ran three simulations with the bulkier model using the limb motion parameters taken from the results of the optimizations with the ‘slimmer’ model (medium—all limbs, medium—forelimbs only, medium—hindlimbs only). Because it is unlikely that substantially different limb motion parameters would give a more effective swimming motion, we did not use optimization to search for new motion parameters. Table 3 shows a comparison of the original (slim) and modified (muscle bulk) model, and for each simulation case gives the swimming distance and vertical deviation from the horizontal. The results showed that the bulkier model was marginally slower in each case, probably due to the increased drag. With both models, however, the contribution of the hindlimbs to locomotion is small. This test shows that manipulation of the fine details of the model does not have a major impact on the swimming speed. Larger modifications to the body shape could have a more substantial effect, and is an area for future work. With larger changes to the body shape, it would be necessary to use optimization to search for the most effective limb motions.

**Table 3:** Comparison between body models. Results from simulations using the original body mesh (slim) and a model with more muscle bulk around the base of the limbs. The model with more bulk travelled a shorter distance, but the relative contribution of the limbs remain the same. The small vertical deviation indicates that all six simulations resulted in straight swimming.

Limbs Used	Distance (m)	Vertical Deviation (m)
All Limbs—Medium—Slim	0.92	0.036
All Limbs—Medium—Muscle Bulk	0.74	0.020
Forelimbs—Medium—Slim	0.83	0.016
Forelimbs—Medium—Muscle Bulk	0.63	0.018
Hindlimbs—Medium—Slim	0.26	0.0021
Hindlimbs—Medium—Muscle Bulk	0.25	0.0056

There is great variation in head and neck proportions, flipper aspect ratios and relative limb proportions within the clade [37]. For example, in *Meyerasaurus* and other Lower Jurassic plesiosaurs the fore- and hindlimbs are subequal in size, whereas in derived pliosaurids the hindlimbs are larger than the forelimbs, and in derived plesiosauroids the forelimbs are largest. Furthermore, *Meyerasaurus* has high aspect ratio flippers, whereas some genera (e.g. *Cryptoclidus*) have low aspect ratio flippers [37]. Head and neck proportions also vary considerably within Plesiosauria. ‘Plesiosauromorph’ taxa possess a long neck and small head, while ‘pliosauromorph’ taxa possess a short neck and large head [2]. The *Meyerasaurus* model used in our experiments possesses an intermediate morphology with a moderately long neck and moderately large skull, so the results represent a generalized plesiosaur morphotype. Variations in head and neck size could shift the center of mass relative to that of our model, possibly affecting the relative contributions of the forelimbs and hindlimbs during swimming. A longer neck and/or a larger head would increase drag and slow the forward motion in a manner similar to our test with increased limb muscle bulk. It is also possible that the tail contributed to forward thrust during swimming. Although we conservatively extend our general conclusions for *Meyerasaurus* to all plesiosaurs, our method could be sensitive to substantial changes in bodily proportions, and different plesiosaurs may have swam in different ways. Further experimentation is therefore required to assess how bodily variation might affect locomotion in other types of plesiosaurs.

## Methods

We simulate the plesiosaur as a set of rigid bodies bound by joints, submerged in inviscid, incompressible fluid flow, governed by the Euler equations [31]. The plesiosaur can move each of its limbs in the fluid by exert torques on each actuated joint. Through the simulation of two-way interaction between the plesiosaur and the fluid, the joint motion will result in both the movement of the fluid and the locomotion of the plesiosaur.

### Fluid simulation

We simulate the fluid dynamics based on Euler equation (Navier-Stokes equation without the viscosity term), since the viscosity of water is negligibly small.

$$\frac{\partial u}{\partial t}+u∙\nabla u+\frac{1}{\rho }\nabla p\;=\;g$$

∇∙u = 0

where ***u*** is the velocity of fluids, *ρ* is the density, *p* is pressure and ***g*** is gravity. We simulate the fluid using a staggered MAC grid [38] based solver. We use BFECC [39] to integrate the advection term and use explicit Euler scheme to integrate the gravity force. We solve the incompressibility term along with the two-way coupling between fluids and solids (See Section 2.3 for details).

### Articulated rigid body simulation

The dynamics of articulated rigid body systems (the plesiosaur) is described by the equations of motion in the generalized coordinate:

$${M(q)\ddot{q}+C(q,\dot{q})\;=\;\tau }_{int}+\tau_{ext}$$

where ***q***, $\dot{q}$ and $\ddot{q}$ are positions, velocities and accelerations in the generalized coordinates respectively, ***M(q)*** is the mass matrix, ***C*** is the Coriolis and Centrifugal force, ***τ***_*ext*_ are the external generalized forces, including the fluid pressure and gravity, and ***τ***_*int*_ are internal torques exerted by the actuated joints. Given a reference swimming stroke, we use Stable Proportional-Derivative controllers [40] to compute the internal joint torques ***τ***_*int*_ to closely track the stroke.

### Two-way coupling between fluids and articulated rigid bodies

We build our two-way coupling solver based on Tan’s two-step procedure [31]. In the first step, both the fluid and the solid are simulated independently. In the second step, a linear system of the pressure field is formulated, taking into account both the incompressibility of the fluid and the dynamics of the solid due to the fluid pressure.

**Fig 7:**
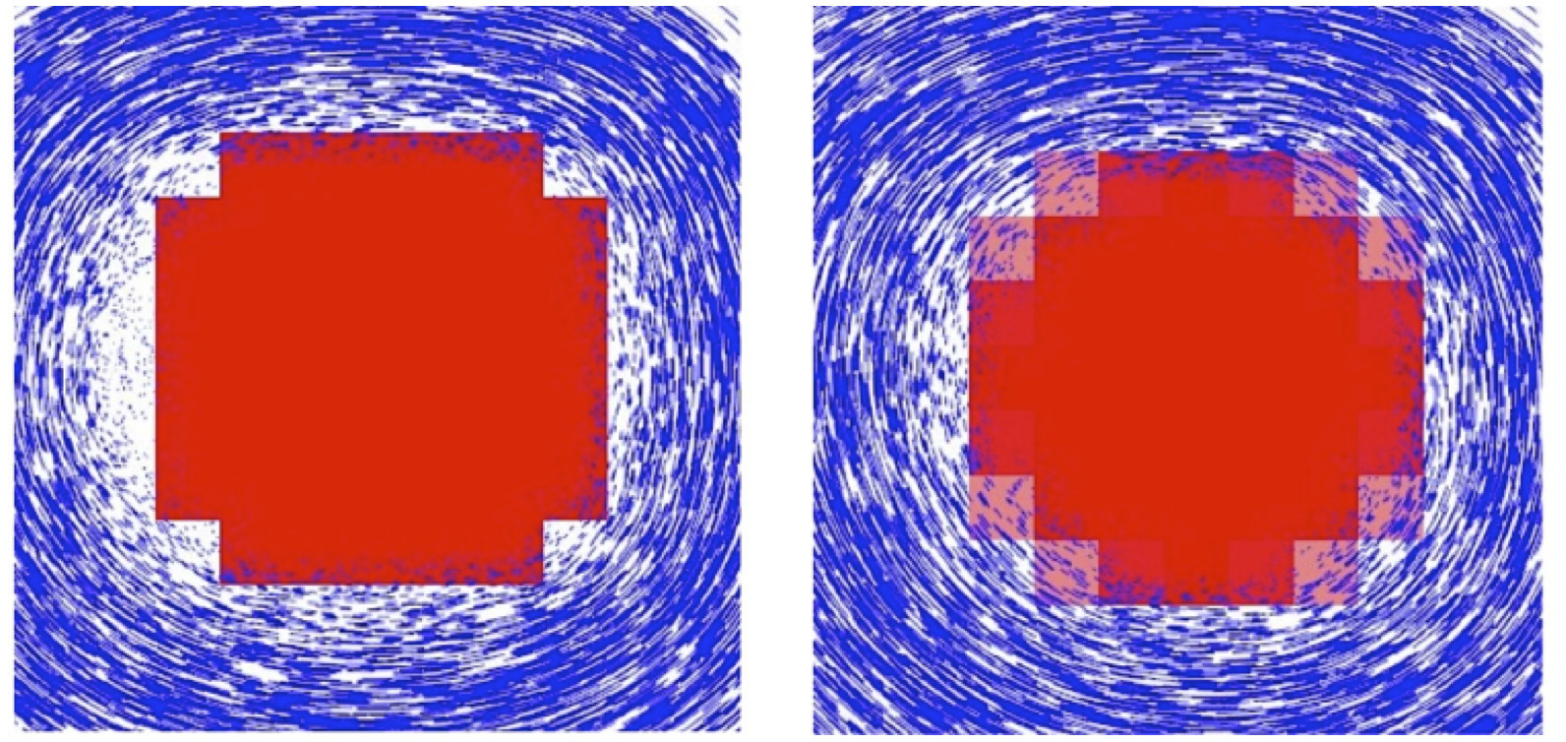
Simulations of fluid surrounding a voxelized circle. In the left 20x20 simulation, due to low resolution, the circle is mapped to a square shaped solid region (red blocks). In the right simulation, 3x3 sub-grid resolution is used, resulting some partially filled fluid grids (transparent red blocks). Notice the round shape of the circle is much better preserved than the left one, which does not use sub-grid resolution.

Our simulator needs to voxelize the plesiosaur onto the grid at each time step, which marks the grid cells that are inside the animal as solid cells and the remaining as fluid cells. Due to the large computational requirement of the two-way coupling simulation, we use relatively coarse grid resolution (100x80x60 to represent a 6.6 by 5.28 by 3.96 m^3^ region of water), which will cause stair-step boundary artifacts at the interface between the fluid and the animal. This can result in seemingly higher viscosity near the animal’s body. For this reason, we incorporate the variational approach [41] into our two-way coupling simulation. This enables us to perform simulation with sub-grid resolution and gain smoother results at the solid-fluid interface with almost negligible additional cost (Fig 7).

The traditional fluid simulation enforces the incompressibility by solving the following Poisson equation, with Neumann boundary condition at fluid-solid faces and Dirichlet boundary condition at free surface.

$$\nabla^{2}p\;=\;\frac{\rho }{\Delta t}\nabla ∙u*$$

Following Tan’s [31] derivation, at coupled faces (solid-fluid interface), the total generalized force exerted by the fluid pressure to articulated rigid-bodies is an integral of pressure over the surface of the plesiosaur:

$$\tau_{total}\;=\;\iint_{S}^{}\tau \;=\;\iint_{S}^{}J^{T}pn\;=\;\sum_{i\;=\;1}^{k}J_{i}^{T}{\left(\Delta x\right)}^{2}p_{i}n_{i}$$

where ***J***_*i*_ is the Jacobian matrix at the ith fluid-surface interface, Δ*x* is the length of a single grid cell, *p* is the pressure inside the fluid cell and ***n*** is the solid surface normal.

Similar to Batty’s variational approach [41], we apply divergence theorem to convert the surface integral to the volume integral.

$$\tau_{total}\;=\;\iint_{S}^{}J^{T}pn\;=\;{∭}_{V}^{}\nabla ∙(J^{T}p)\;=\;{∭}_{V}^{}J^{T}(\nabla p)+\nabla ∙(J)p$$

We dropped the second term in the integral because the divergence of ***J*** is always zero for rigid body motions, and since *p* is zero everywhere inside solids and equals to the pressure in fluid cell at fluid-solid interface, we can discretize the above integral to be

$$\tau_{total}\;=\;{∭}_{V}^{}J^{T}(\nabla p)\;=\;\sum_{i\;=\;1}^{k}{v_{i}{\left(\Delta x\right)}^{3}J}_{i}^{T}(\frac{p_{i}}{\Delta x})n_{i}\;=\;\sum_{i\;=\;1}^{k}{v_{i}{\left(\Delta x\right)}^{2}J}_{i}^{T}p_{i}n_{i}$$

where *v*_*i*_ is fluid volume fraction at the coupled interface.

The acceleration in the generalized coordinate of the coupled interface is

$$\ddot{q}\;=\;M^{-1}\tau_{total}$$

where ***M*** is the mass matrix of the articulated rigid-body. Therefore the acceleration for the coupled interface in Cartesian space would be

$$a\;=\;n^{T}\left(\dot{J}\dot{q}\right)\;=\;n^{T}\left(J\ddot{q}+\dot{J}\dot{q}\right)\;=\;n^{T}(JM^{-1}{∭}_{v}J^{T}\left(\nabla p\right)+\dot{J}\dot{q})=\;n^{T}\left(\sum \begin{array}{l} k\\ i\;=\;1 \end{array}v_{i}{\left(\Delta x\right)}^{2}JM^{-1}J_{i}^{T}p_{i}n_{i}+\dot{J}\dot{q}\right)$$

Intuitively, the variational approach adds fluid volume fraction information *v*_*i*_ to the original coupled linear equation [31], making use of volume fraction weighted stencils, instead of being discrete values of zero or one.

To compute the fluid volume fraction *v*_*i*_, we perform inside-outside tests of the plesiosaur model at the center of every cell of a higher resolution grid. We shoot a ray at the cell center and count the number of intersections (parity) between the ray and the model. Since our model is watertight, if the intersection count is even, the cell is outside of the body of the plesiosaur, and thus occupied by the fluid. Otherwise, the cell is inside the creature, and thus it is solid. We implemented a Surface Area Heuristic Kd-Tree [42] to accelerate ray-model intersection performance. In our implementation, we use a sub-grid resolution of 3x3x3 to compute the fluid volume fraction, achieving 1/27 volume fraction precision in the pressure solve.

Finally, we construct a sparse symmetric positive linear system with the following equations at the fluid-solid faces,

$$D(\sum_{i\;=\;1}^{k}v_{i}{\left(\Delta x\right)}^{2}JM^{-1}J_{i}^{T}p_{i}n_{i})\;=\;D(\frac{u^{*}}{\Delta t}-\dot{J}\dot{q})$$

where ***D*** is discretized volume-weighted divergence operator and u* is the intermediate velocity field before enforcing incompressibility. We use normal stencils of Laplacian and Divergence operators at fluid-fluid faces. We apply Preconditioned Conjugate Gradient solver to solve the system, after which we project the fluid velocity with the following equation

$$u\;=\;u^{*}-\Delta t\frac{\nabla p}{\rho }$$

where u* and ***u*** represents the fluid velocity before and after projection. As a result, we simultaneously enforce incompressibility of the fluids, compute dynamics of the solids, satisfy boundary velocity constraints and achieve smoother fluid flow at the solids-fluids interface.

**Fig 8:**
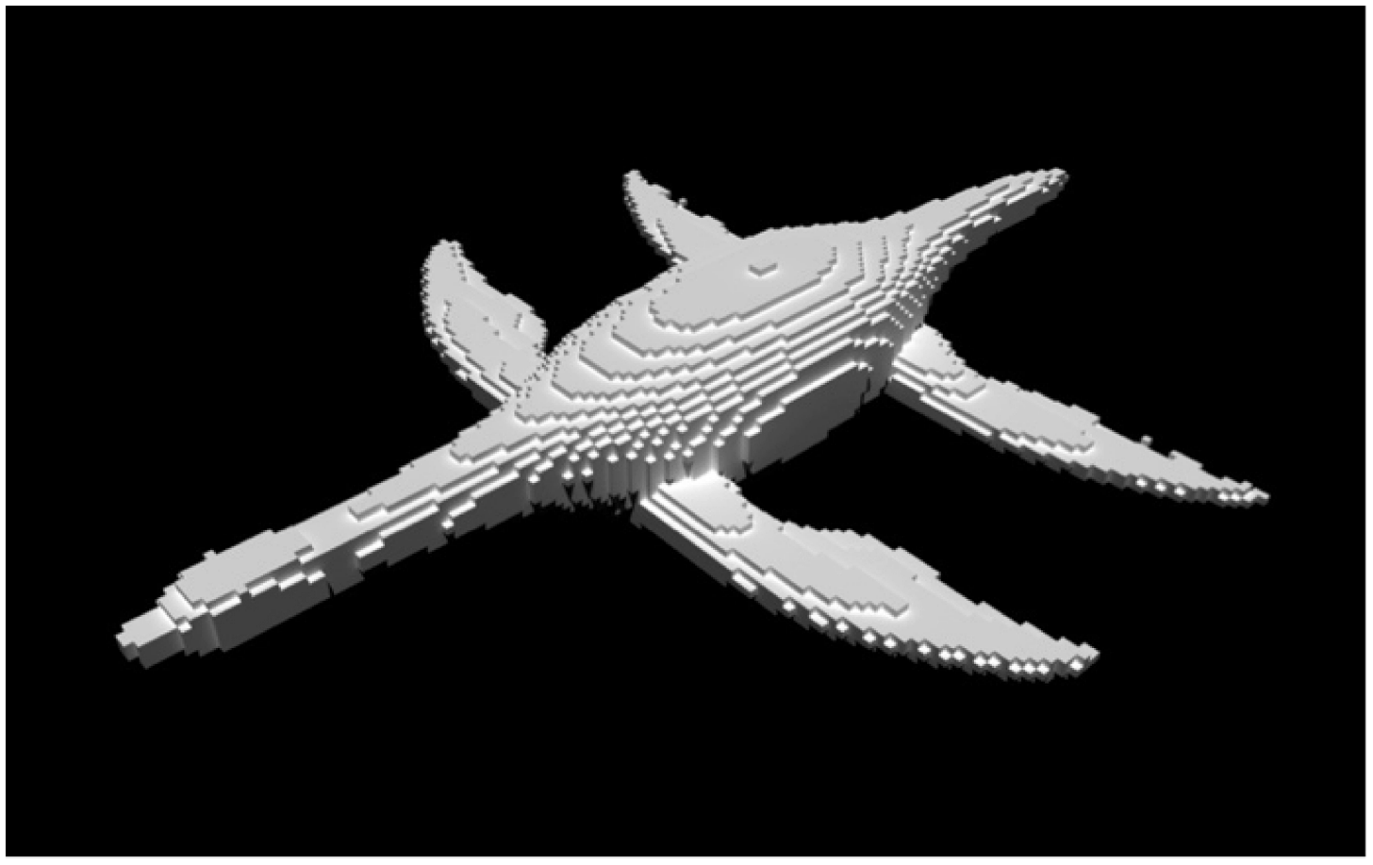
Model voxelization. The voxelized version of the plesiosaur mesh, using 3x3x3 sub-grid voxelization. The model is re-voxelized at each simulated time-step, which further minimizes the effects of voxelization.

Note that our simulated water does not behave exactly the same as real water. Two common issues in numerical fluid simulations, numerical viscosity and voxelization artifacts, affect the accuracy of our two-way coupled simulator. The numerical errors in the simulation can make the simulated fluid more viscous than its real counterpart. This is called numerical viscosity [43]. Even though we dropped the viscosity term from the Navier-Stokes equations and used a higher-order integration scheme, BFECC [39], the numerical viscosity cannot be eliminated entirely. Voxelization artifacts are caused by converting the rigid bodies (the plesiosaur) into a regular grid of cells for fluid simulation (Fig 8). The streamlined body shape of the animal is lost in this voxelization process. As a result, the animal may swim slower than in real life due to the increased form drag [44]. The variational framework [41] that we used allows us to simulate at sub-grid resolutions, and thus ameliorates this problem. However, this issue cannot be completely eliminated.

### Optimizing swimming strokes with Covariance Matrix Adaptation (CMA)

Given a plausible range of motions for the front and back limbs, we wish to find the swimming motion that propels the animal the farthest distance in a given amount of time. We also want to favor straight swimming. Since a single swimming motion requires the specification of 26 parameters we formulate our question in terms of optimization. Let us call a specific set of swimming motion parameters a motion sample, and we can think each such motion sample *s* as a single point in a 26 dimensional space. Given a motion sample, the plesiosaur/fluid simulator generates a swimming motion through the hydrodynamic interaction. We formulate the quality function *q*(*s*) that favors a swimming stroke that leads to a longer swimming distance, and we penalize deviation from swimming straight.

We calculate the swimming distance as the displacement of the plesiosaur’s center of mass (COM) in one swimming cycle projected onto its initial heading direction. The deviation from straight swimming is decomposed into two components: Directional deviation is measured by the displacement of COM that is perpendicular to the initial heading direction. Orientation deviation is measured simply as the orientation change in one swimming cycle. Since the simulation starts with a static plesiosaur submerged in motionless water, we evaluate the objective function only after the first stroke cycle is completed, when the plesiosaur has reached a steady speed. Forelimb-only and all limb optimizations were initiated with the front limbs in the fully abducted position and the hindlimbs in the neutral position. Hindlimb-only optimizations were initiated with the hindlimbs in the fully abducted position and the forelimbs in the neutral position. This forced all of the gait samples to begin with a downstroke, so that no gait was penalized for a slow start that is mid-way through a stroke.

We used Covariance Matrix Adaptation (CMA) [34, 45] to search for the sample *s* that gives us the highest quality. CMA is a sample-based approach to optimization that uses a Gaussian distribution to guide its selection of motion samples to test. Each iteration of the CMA draws a new set of samples based on the current distribution, and evaluates the quality of these samples using *q*(*s*). The mean and the covariance matrix of the Gaussian are then updated according to a subset of sample with higher quality. As more iterations are taken, the covariance matrix narrows down towards the best quality sample. In our case, the best sample is the swimming motion parameters that move the simulated plesiosaur the farthest in a straight line through the water.

To converge to the optimal motion, CMA requires many samples to be evaluated. Each sample evaluation requires a full simulation of the specified motion, and each such plesiosaur/fluid simulation requires roughly one hour of computation. We used 31 samples per iteration of CMA, and we found that a typical optimization run converged in about 70 iterations, after which the quality of the motion samples does not improve. This means that a full optimization requires more than two thousand swimming simulations. We made use of a compute cluster with 32 nodes, so that the computations for a single iteration were all calculated in parallel on separate nodes. (One of these computer nodes coordinates the simulations of the other 31 nodes.) Even with the compute cluster, performing CMA optimization for a single set of joint ranges requires three days to run.

Although CMA converges to high quality samples, there is no guarantee that it will find the best possible sample in our 26-dimensional search space. This explains why, for the wide joint range, the best forelimb-only swimmer that was found is slightly faster than the all-limbs swimmer (Fig 5). To verify that this was due to small variations between optimization runs, we ran a total of three optimization runs for each of these two cases for the wide joint range. The three optimization runs for all-limbs gave speeds of 0.745, 0.75, and 0.785 (m/s), and the three runs for forelimbs-only resulted in speeds of 0.745, 0.80, and 0.84. Note that these ranges overlap. Fig 5 reports the fastest speeds from each of these sets of three optimization runs.

## Supporting Information

S1 Video Efficient swimming motions. Animation showing the most efficient swimming motions that were found in each of the three joint ranges (wide, medium, and narrow). For each joint range, animations are shown for both sets of limbs, for only forelimbs, and for only hindlimbs. White streamlines show the motion of the surrounding fluid.
(MP4)

S2 Video Tip traces. Animation showing the motion of the tips of the limbs for the most efficient swimming motions in each of the three joint ranges. The camera view is locked to move with the plesiosaur model to more clearly show these tip traces. For each swimming motion, both a lateral view and a posterolateral view are shown.
(MP4)
